# The Perceived Social Burden in Celiac Disease

**DOI:** 10.3390/diseases3020102

**Published:** 2015-06-19

**Authors:** Carolina Ciacci, Fabiana Zingone

**Affiliations:** Celiac Center, AOU San Giovanni di Dio e Ruggi D’Aragona, University of Salerno, Salerno 84081, Italy; E-Mail: fabiana.zingone@outlook.com

**Keywords:** gluten free diet, symptoms, social, burden, psychological, adherence

## Abstract

In the present paper, we discuss the change in celiac disease (CD) awareness and perception through patients’ concerns and the most recent literature. Nowadays CD has moved in the public awareness (both doctors and population) from a rare disease to a common one and the gluten free diet (GFD) is no longer the exclusive therapy for CD patients but is becoming a popular health choice for everybody. Gluten-free food, once hard to find and requiring home preparation, is now available at restaurants and grocery stores. However, the quality of life of those affected by CD seems to be still compromised and this is particularly true for those who find it difficult to adhere to a GFD and those who were asymptomatic at the time of diagnosis. Intervention at diagnosis and follow-up to improve the patients’ adaptation to the condition and its limitations should be implemented.

## 1. Introduction

In the last decades, the celiac disease (CD) epidemiology [1,2] and awareness [3,4] have been changing. CD is now considered a common disease and the gluten free diet (GFD) is no longer the exclusive therapy for CD patients but is becoming a popular health choice for everybody [5,6].

In the past, CD was usually diagnosed in the presence of the “classical triad”: chronic diarrhea, abdominal distention, and failure to thrive [7]. A celiac patient was then likely a child and the family felt lonely to face this “strange” disease with an odd dietetic therapy. In the latest decades, CD has been recognized as a worldwide problem, affecting people of all ages and characterized by a wide spectrum of clinical presentations [8,9]. We learnt that CD has many faces and screening programs with sensible and specific serological markers showed that CD is present in different conditions and also in apparently healthy people [8]. We also learned that CD is not only more easily recognized but also increasing in its prevalence [2,10]. All over the world we are facing what can be defined as a celiac epidemic.

CD has moved in the public awareness (both doctors and population) from a rare to a common disease. Gluten-free food, once hard to find and often requiring home preparation, is now readily available at restaurant and grocery stores.

Typing the words *celiac disease* in Google generates more than 6,800,000 results in few seconds. Millions of news articles and non-scientific papers provide large quantities of information, which influence healthcare policies more than scientific papers alone can do [11].

The perception of CD has changed, it is no longer a “disease” but a condition, *id est* a factor affecting the way people live or work, especially with regard to their well-being. The GFD is not only the sole therapy for CD patients but also a choice for non-CD patients [6].

In this paper, we examined the following factors involved in the perceived quality of life (QoL) of a CD patient:

1. Awareness of the disease among caregivers;
2. Social burden;
3. Quality of life (QoL) and coping strategies.

We selected some of the comments made by the patients during outpatients visit as an example of the most challenging situations and used these as a guide in the literature search. We used the PUBMED database and the search terms “celiac disease” AND “quality of life” (240 hits) “burden” (68 hits), “awareness” (165 hits), with the final search performed in May 2015. Systematic reviews, case series, case-control studies, and randomized controlled clinical trials were analyzed and chosen as a support of this opinion paper.

## 2. Awareness of the Disease among Caregivers

“I feel more reassured if my doctor knows well my disease”

L.T. 45 years old.

To our knowledge, there is no paper that directly deals with physician knowledge of CD. The following studies, however, suggest that active case-finding, presupposing a good knowledge of the disease presentation, is not a common practice among physicians, A study from 2005 showed the need for education among Californian GPs, as only 35% of them had ever diagnosed CD [12]. In Europe the picture is variable. While in France CD awareness is quite low among GPs and the diagnosis is much better recognized by gastroenterologists and pediatricians [13], in Finland there is an increasing awareness among GPs who are now in charge of the diagnosis. The passage from tertiary centers to GPs has been shown to be a good strategy to reduce the time lapse between onset of symptoms and diagnosis [14]. Case–finding strategies conducted by GPs in USA and Italy have showed that awareness campaigns among doctors improve the knowledge of CD and its diagnosis [15,16]. Catassi *et al.* [17] showed in a large screening study that the CD diagnoses were only the tip of an iceberg and the majority of CD cases were under the water level. Recently, Zingone *et al.* [18], after more than ten years from the Catassi’s study, have showed that CD incidence in Benevento, a rural area of Campania, is lower compared to other nearby provinces, assuming that residents there tend to visit their doctors only in case of severe clinical conditions making it more difficult to capture subclinical and non-classic forms of CD. Similarly, a study conducted using GP datasets in the UK found that children living in less socioeconomically deprived areas were about twice as likely to be diagnosed as those from more deprived areas [19]. This latter finding may be due to the fact that underprivileged people may be less likely to seek medical care or consultation in general and thus be potentially less likely to be tested for CD. However, another possibility is that underprivileged people have an inferior hygienic environment that may protect against celiac disease [20]. After diagnosis is made, however, the follow-up is made in most cases by a specialized Celiac Center in which the CD awareness is assured. Follow-up should include a dietitian consultation, as patients report better satisfaction when they are referred to dieticians [21,22] and psychological support when required [23]. The importance of a psychological support has been recently reported by Sainsbury *et al.* [24] who found that psychological symptoms and the GFD adherence were more strongly related to reduce QoL than gastrointestinal symptoms. Despite the large mass of information from media, the CD awareness among doctors can be improved to enable early diagnosis and to support the patients once the diagnosis is made.

## 3. Social Burden

“When I go out with my friends I feel different from them… there is no restaurant in my little town that serves gluten free food”

F.G 25 years old.

Patients with CD feel cautious when eating food not prepared at home. The unavailability of gluten-free food outside home and the low CD awareness among food workers have been a problem in the past years, although they are rapidly changing. A survey conducted in 2003 among chefs in the UK showed that they knew less about CD than the general public [25]. The authors conducted the same survey in 2013 [26], which showed a significant increase in chefs’ awareness of gluten-related disorders (both CD and non-celiac gluten sensitivity) and a similar level of recognition of the gluten-free symbols among the public and chefs (about 40%).

A 2006 survey conducted on 2681 adult members of the Canadian Celiac Association showed that 44% of them found difficulties following the GFD which included: determining if foods were gluten free (85%), finding gluten free foods in stores (83%), avoiding restaurants (79%), and avoiding travel (38%) [27]. Another survey conducted in Canada seven years later among the same population showed that difficulties and negative emotions were experienced less frequently by those on the diet for >5 years, although food labeling and eating away from home remained very problematic [28].

Similarly, a survey aimed at evaluating the adherence to GFD in children and adolescents showed that participants reported good adherence at home and school, but low adherence at social events. Children reported that the main significant barriers to a good adherence to the GFD were availability of gluten free foods, labeling of food and their cost [29]. Roma *et al.* reported the answers of 73 children questioned about the main causes of non-compliance and the most frequent reasons were: poor palatability (32%), dining outside home (17%), poor availability of products (11%), and asymptomatic disease diagnosed by screening (11%) [30].

The parental knowledge of CD is also important for a good diet adherence and a better disease awareness of their children. A recent study conducted in the UK among parents of CD children at the time of diagnosis reported that 98% of the parents understand that GFD is the treatment of CD and 95% know that the GFD is for life. However, specific dietary knowledge was lacking with only one-third of the parents correctly identifying all the gluten containing food. The internet was the most common source of information (70.6%) [31]. Higher levels of parenting stress has been observed in parents of CD children compared to parents of healthy children [32].

Patients’ support groups have played a relevant role in the last decades, organizing special social events for patients and families and providing education to patients, doctors, dietitians and the food service industry.

The most recent and unique intervention in this matter is a randomized trial showing the efficacy of an interactive online intervention to improve the QoL and the GFD adherence [33].

However, beside the external help, Ford *et al.* reported that personal tools such as self-efficacy and illness perception are influential factors and play a role in the individuals’ psycho-education. A targeted therapeutic intervention might improve GFD adherence and enhance psychological well-being for CD patients [34].

In conclusion, the published studies indicate that, although there has been a positive change over time, there is still the need for additional training and education about CD and GFD [35].

## 4. Quality of Life and Coping Strategies

The question is: Has the CD QoL improved now that at school, at work, at restaurant the words “gluten free diet” very often do not require any other information to be understood?

A review of the literature over time tells us that QoL of a CD patient before/at diagnosis is strongly influenced by the presence of gastrointestinal or non-gastrointestinal symptoms. Differently, after the diagnosis, the QoL of a CD patient is mainly related to the difficulty of having a chronic condition and the limitations imposed by the GFD as well as the difficulties in maintaining a good diet compliance [36,37]. Poor dietary adherence has been often associated with a poor QoL [38,39] but it is difficult to identify which is the cause and which the effect.

“*When I am at a restaurant with my friends, I often eat food with gluten because I do not want to be different*”

G.G. 25 years old.

Celiac patients’ bad dietary adherence might be caused by the difficulty to accept their condition and the limitations that it imposes. A study conducted in 2001 showed that adult celiac patients reported inability to communicate feelings and a tendency to conformist behavior leading to a lifestyle limited by a lifelong condition [40]. At that time, patients feared that social contexts such as restaurants or convivial gatherings are occasions in which they may be identified by others as disease-affected, resulting in reputational damage. There are contradictory results about the relationship between dietary compliance and QoL. A 2010 long-term longitudinal study suggested that subsequent deterioration in QoL was associated with a lack of dietary adherence [41]. Other studies reported no differences in QoL scores between patients with full adherence and patients with partial/non adherence to GFD [42,43]. In particular, Barratt *et al.* reported that the perceived difficulty of adhering to a GFD and not the correct adherence to that may be associated with a decline in QoL [42].

“I do not want to go to my friends’ birthday parties because I do not want them to say that I cannot eat the cake because I am ill”

CM, 12 years old.

Looking at adolescents, Wagner *et al.* [44] found that bad compliance to the GFD was the major cause of low general QoL. Non-compliant adolescents had more physical problems, a higher burden of illness, higher feelings of “ill-being” and more family problems than GFD compliant adolescents. Moreover, in these patients, a late CD diagnosis was associated to more problems at school and in social activities. A school-based cross-sectional screening study recently found that children with undetected CD reported comparable health-related QoL to CD children on a GFD and those without CD [45]. Finally, a prospective study on screening-detected CD children described that ten years after diagnosis, the QoL of the children with CD was similar to that of the reference population [46].

A qualitative study, using a focus group interview, investigated QoL in children and adolescents with CD to identify patient concerns in living with CD and on a GFD [47]. Two main categories were identified: “having CD” (composed by the diagnosis-process, self-perception, awareness of CD, social and emotional impact of CD, and thoughts about the future) and “coping with CD” (coping with food and coping with social situations). Several ways to alleviate stress were identified in the coping group: comparing CD to other chronic diseases, believing they were healthier than others as they did not eat as much unhealthy food, accepted their diagnosis and came to the conclusion that “there is nothing to be done about it” [47]. Living with CD is perceived as requiring several sacrifices which can influence the QoL in children on a GFD [48].

“*I was so ill before diagnosis, I daily had diarrhea, abdominal pain, headache… now I am a new person, I love the GFD*”

R.T. 45 years.

The GFD induces an improvement in QoL in symptomatic patients, while a similar effect has not been clearly reported in patients with subclinical/asymptomatic CD. A 2011 Finnish study [49] reported no change of the QoL in screen-detected patients on GFD and another Finnish study [50] in the same year described the QoL improved in symptomatic and in screen-detected symptomatic patients but not in screen-detected asymptomatic patients. Recently, Kurppa *et al.* [51] have described, using a randomized study, that asymptomatic CD patients benefited from the GFD for anxiety and better health, but not for social function when compared to similar CD patients following a gluten-containing diet. Finally, Paarlahti *et al.* [52] reported that a long duration of symptoms before diagnosis, psychiatric, neurologic or gastrointestinal co-morbidities and persistent symptoms were predictors of a reduced QoL.

Apart from the symptoms commonly attributed to CD, other less explored complaints are known to improve on GFD and may have a positive effect of the QoL of patients. For example, sexual activity, which is hardly investigated dealing with a celiac patient, has been shown to be poor, unrelated to gastrointestinal symptoms, and to improve after one year of GFD [53].

Another problem that is likely underestimated in celiac patients is the presence of eating disorders that are more frequent in untreated celiac women, dependent on the presence of gastrointestinal symptoms, and also improve on GFD [54]. Moreover, Zingone *et al.* [55] reported a reduced quality of sleep in CD patients both at diagnosis and on GFD compared with controls. A direct correlation between the quality of sleep and mood disorders and QoL was observed.

## 5. Conclusions

In conclusion, the review of published papers reveals a noticeable increased knowledge of CD among doctors and the wider society. Despite this, the literature search indicates that celiac patients still present some vulnerability in the adaptation to a chronic condition, in particular in their social life. Planned intervention at diagnosis and follow-up to improve the patients’ adaptation to the condition and its limitations [56] and as a consequence their QoL has been suggested.

It should be noted, however, that large controlled follow-up studies on the effect on QoL of any intervention, such as psychological support and repeated dietetic consultation, are still lacking.
